# Severe bleeding from esophageal varices resistant to endoscopic treatment in a non cirrhotic patient with portal hypertension

**DOI:** 10.1186/1749-7922-3-24

**Published:** 2008-07-21

**Authors:** Roberto Caronna, Mario Bezzi, Monica Schiratti, Maurizio Cardi, Giampaolo Prezioso, Michele Benedetti, Federica Papini, Simona Mangioni, Gabriele Martino, Piero Chirletti

**Affiliations:** 1 Department of Surgery "Francesco Durante" – General Surgery N, Sapienza University of Rome, Viale del Policlinico, 00161, Rome, Italy; 2 Department of Radiology, Sapienza University of Rome, Viale del Policlinico, 00161, Rome, Italy

## Abstract

A non cirrhotic patient with esophageal varices and portal vein thrombosis had recurrent variceal bleeding unsuccessfully controlled by endoscopy and esophageal transection. Emergency transhepatic portography confirmed the thrombosed right branch of the portal vein, while the left branch appeared angulated, shifted and stenotic. A stent was successfully implanted into the left branch and the collateral vessels along the epatoduodenal ligament disappeared. In patients with esophageal variceal hemorrhage and portal thrombosis if endoscopy fails, emergency esophageal transection or nonselective portocaval shunting are indicated. The rare patients with only partial portal thrombosis can be treated directly with stenting through an angioradiologic approach.

## Background

Recent advances in interventional radiology, especially the introduction of endovascular portosystemic shunts, have brought about rapid changes in therapy for the complications of portal hypertension [1]. Although the preferred treatment for a patient with variceal bleeding related to portal hypertension remains endoscopic sclerotherapy, when this option fails, as it does in about 15% of the cases, the only alternative is an emergency portosystemic shunt [2]. A surgical shunt procedure is indicated in patients with Child-Pugh A cirrhosis, the transjugular intrahepatic portosystemic shunt (TIPS) in those with Child-Pugh B-C cirrhosis scheduled for liver transplantation [3]. The treatment of variceal bleeding raises different problems in cirrhotic and non cirrhotic patients with portal vein thrombosis. Whereas from 0.6 to 2.6% of patients with cirrhosis have spontaneous portal thrombosis, those without cirrhosis generally do not (Table 1) [4]. The major causes of portal thrombosis in these patients are hereditary or acquired coagulation defects and local factors that include intraabdominal infections (in particular close to hepatic hilum) and surgical or traumatic portal vein damage.

**Table 1:** Etiology of Portal Vein Thrombosis (modified from Sobhonslidsuk A.) [4]

**Thrombophilic Disorders**	**Local factors**
**Inherited disorders**	Infections/inflammation
High risk of thrombosis (low prevalence):	Neonatal omphalitis
**Antithrombin **III deficit	Appendicitis
Protein C deficit	Diverticulitis
Protein S deficit	Pancreatitis
	Cholecystitis
Low risk of thrombosis (high prevalence):	Perforated peptic ulcer
Leiden V factor mutation	Tuberculous lymphadenitis
Factor II mutation	
**Acquired disorders**	**Portal vein injury**
Malignancy	Surgical shunts
Myeloproliferative disorders	Splenectomy
Use of oral contraceptives	Abdominal surgery
Antiphospholipid syndrome	Liver transplants
Pregnancy and postpartum	Blunt trauma
Paroxysmal nocturnal	
hemoglobinuria	
**Mixed disorders**	**Cancer of the abdominal organs Cirrhosis**
Hyperhomocysteinemia	

We describe a case of severe recurrent hemorrhage from esophageal varices in a patient without cirrhosis who had undergone open cholecystectomy 12 months earlier. The failure of endoscopic therapy raised complex problems in deciding how to manage portal hypertension.

## Case presentation

A 58-year-old non alcoholic patient was admitted to hospital for investigation of hematemesis and melena. Twelve months earlier he had undergone elective open cholecystectomy for chronic calculous cholecystitis complicated by a biliary fistula that had resolved spontaneously. An esophagogastroduodenoscopy disclosed bloody esophageal varices and the bleeding was successfully controlled by endoscopic sclerotherapy. During repeated endoscopic sessions the varices were progressively eradicated by ligation. Laboratory serum screening and liver ultrasound excluded the presence of cirrhosis while the portal echo-color Doppler ultrasound examination detected portal vein thrombosis. Laboratory tests of blood coagulation yielded normal findings.

Three months after the latest endoscopic follow-up, the patient was readmitted to hospital with severe hemorrhage. A repeat endoscopy showed recurrent variceal hemorrhage that responded poorly to sclerotherapy leaving intermittent bleeding. Because endoscopic therapy failed to control the bleeding and portal thrombosis made TIPS unfeasible, an open surgical procedure was scheduled (nonselective side-to-side portacaval shunting). At operation, because severe fibrosis prevented access to the hepatic hilum thus making nonselective portacaval shunting unfeasible, the patient underwent an alternative surgical procedure, esophageal transection. After surgery the bleeding continued. A second portal echo-color Doppler ultrasound study and computed tomographic (CT) scan disclosed a thrombus completely occluding the right portal vein branch with an apparent re-channeling of the left branch. An emergency angioradiologic procedure was scheduled.

**Figure 1:**
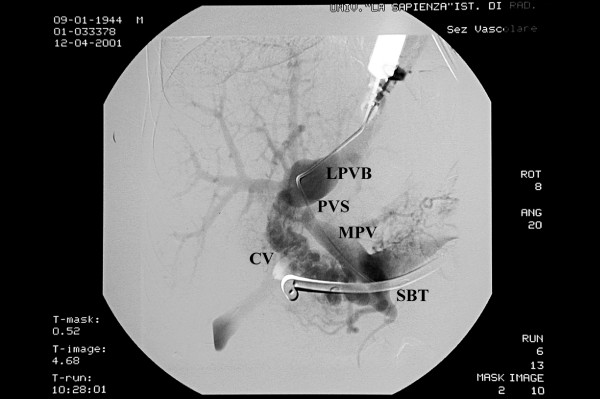
**Percutaneous transhepatic portography: the main portal trunk is patent with a tight stricture of left portal vein branch with a poststenotic dilatation**. PVS: portal vein stenosis. LPVB: left portal vein branch. MPT: main portal trunk. CV: collateral vessels. SBT: Sengstaken-Blakemore tube.

**Figure 2:**
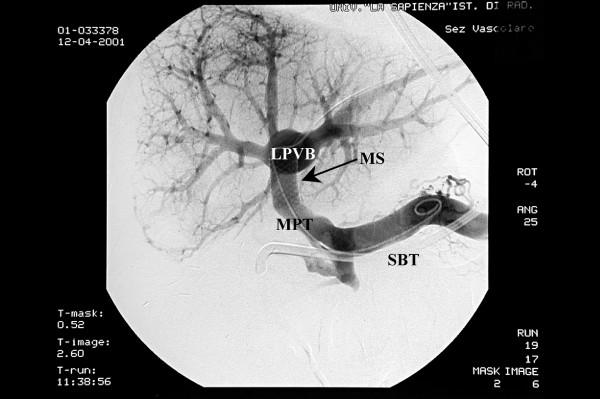
**Percutaneous transhepatic angioplasty of the stricture with a baloon catheter**. A metallic stent has been placed at level of the stricture with disappearance of collateral vessels and improved filling of intrahepatic portal branches. MS: metallic stent. LPVB: left portal vein branch. MPT: main portal trunk. SBT: Sengstaken-Blakemore tube.

A peripheral portal vein branch of the left lobe was punctured under ultrasound guidance with a 22 gauge Chiba needle and a 0.018 inch guide wire was advanced into the left portal vein branch. The transhepatic tract was widened to advance a 4 French cobra catheter into the vein over a 0.035 hydrophilic guide wire. The guide wire was negotiated into the main portal trunk and a direct portographic scan was obtained. Portography showed a patent main portal trunk, a tight stricture between the portal vein and the left branch, with evidence of a post-stenotic dilatation. Collateral vessels along the hepatoduodenal ligament allowed for revascularization of the right portal branch (Figure 1). The portal venous pressure was 32 cm H_2_O in the portal vein before the stricture and 12 cm H_2_O in the left portal branch distal to the stricture, with a gradient of 20 cm H_2_O. The stricture was dilated with an 8 mm angioplasty balloon catheter and a metallic stent (30 mm long and 10 mm in diameter) was placed at the level of the stricture (Figure 2). After stent deployment the stenotic area was further dilated with a 10 mm angioplasty balloon catheter. After the procedure the pressure gradient across the stricture was 6 cm H_2_O. Post-procedural portography showed that the stricture had disappeared and filling of the intrahepatic branches had improved. The collateral vessels along the hepatoduodenal ligament had disappeared as well, owing to the reduced portal pressure gradient.

The bleeding subsequently stopped and at a recent follow-up examination (seven years later) the patient had no signs or symptoms of esophageal varices and of stent obstruction.

## Discussion

Although biliary complications can occasionally develop after open cholecystectomy the risk is considerably lower after open surgery than after a laparoscopic procedure (0.4% vs 1.6%) [5]. Recently reported complications include associated vascular and biliary injuries [6] but no reports have described portal vein lesions similar to those in our patient. The most important problem in our case was the emergency treatment of bleeding esophageal varices in a patient with portal hypertension secondary to portal vein thrombosis. Although the current standard first approach is generally endoscopic (sclerotherapy or ligation) this procedure has a reported failure rate of 15% [7]. In patients without cirrhosis, in whom portal hypertension is related to thrombosis or portal stenosis, failed endoscopic treatment of variceal bleeding raises unusually complex problems of management. In general, good liver function permits good control bleeding (normal coagulation indexes and a normal platelet count). In addition, the development of portal cavernomatosis often tends ultimately to invert the hepatoportal blood flow from hepatofugal to hepatopetal thus reducing the risk of esophageal variceal bleeding. But if the bleeding continues after endoscopy, the patient should undergo an emergency angioradiologic procedure or surgery. Recent studies describing percutaneous endovascular treatment of portal stenosis with or without stenting in patients (especially children) who have undergone liver transplantation [8,9] report an unsuccessful outcome related to portal vein occlusion in about 25% of the patients even in those who underwent elective procedures. The only patients with portal hypertension who should currently undergo emergency surgery for variceal or congestive gastropathy bleeding are those with Child-Pugh class A cirrhosis not scheduled for liver transplantation and those without cirrhosis with portal obstruction. The standard emergency surgical options are esophageal transection and nonselective portosystemic shunting [7] if the prestenotic main portal trunk is available. Although esophageal transection has the disadvantage of a relatively high incidence of early rebleeding, it is associated with a lower incidence of hepatic encephalopathy than portosystemic shunting, a more complex surgical procedure that nevertheless results in better control of bleeding [10]. The mortality rate for these two operations reaches 25% in emergency, and is related more to the associated liver failure than to the procedure itself.

Although Japanese investigators have suggested that patients with portal thrombosis should undergo esophageal devascularization according to Sugiura [11] a US experience reports a percentage of postoperative recurrent bleeding ranging from 35 to 50% [12].

Our patient's continued bleeding and the unsuccessful sclerotherapy necessitated emergency surgery (esophageal transection). After surgery failed we decided on a percutaneous endovascular approach, which documented a patent, but angulated and stenotic left branch of the portal vein. Portography showed that TIPS (inserting the stent between the median suprahepatic vein and the left intrahepatic branch of the portal vein) was unfeasible because the excessive angulation of the left branch seemed unlikely to guarantee stent patency and would inevitably have caused severe hepatic encephalopathy. By performing an angioplasty and placing a metallic stent we reduced the portal pressure and stopped the bleeding, thus restoring the hepatic portal blood flow.

## Conclusion

Patients who present with esophageal variceal hemorrhage and portal thrombosis are first managed by endoscopic therapy. If endoscopy fails and a TIPS is not feasible owing to portal thrombosis the patient should undergo an emergency surgical procedure. Esophageal transection is preferred because it is relatively simple, rapid and less likely to cause hepatic encephalopathy. If esophageal transection fails then the only alternative is surgical portacaval shunting, a complex procedure whose success depends crucially on the site, extension and pathogenesis of the portal thrombosis. In patients, such as ours, who have only partial portal thrombosis an angioradiologic transhepatic procedure with or without stenting can control portal hypertension.

## Competing interests

The authors declare that they have no competing interests.

## Authors' contributions

RC conceived of the study, wrote the manuscript and partecipated to surgical procedures and to preoperative and postoperative patient management, MB performed all radiological procedures, MS partecipated in manuscript design and coordination, MC partecipated to surgical procedures and to preoperative and postoperative patient management, GP partecipated to surgical procedures, MB partecipated in manuscript design and coordination, FP partecipated in manuscript design and coordination, SM participated in licterature reviewing and patient follow-up, GM participated in licterature reviewing and patient follw-up, PC was the first operator, is the Chief of the Surgical Unit and participated in manuscript design. All Authors read and approved the final manuscript.
